# Study on Dielectric Properties of Nanoclay-Modified Disulfide-Containing Polyurea Composites

**DOI:** 10.3390/nano16030171

**Published:** 2026-01-27

**Authors:** Xinjian Li, Fan Wang, Haowen Yin, Yang Wang, Guangxi Li, Junjie Huang, Yanhe Yuan, Minghao Zhou, Shuai Zhao, Yingjie Liang, Guangyu Cao, Le Li

**Affiliations:** 1 Lianyungang Power Supply Company, State Grid Jiangsu Electric Power Co., Ltd., Lianyungang 222000, China; 18730980186@163.com (X.L.);; 2 Hebei Key Laboratory of Green and Efficient New Electrical Materials and Equipment, North China Electric Power University, Baoding 071003, China

**Keywords:** insulating materials, dielectric properties, extreme weather, wind deviation environment, polyurea, breakdown strength

## Abstract

To address the frequent faults (e.g., bird-related hazards, wind deviation) of transmission lines under extreme environments and the limitations of traditional insulating materials (insufficient dielectric properties, poor interface compatibility, etc.), this study synthesized a disulfide-containing polyurea (DPU) with dynamic covalent bonds and prepared Halloysite nanotubes (HNTs) modified by aminopropyltriethoxysilane (APTES) to form the HNTs/DPU composite. Methods included characterizations like Fourier transform infrared spectroscopy (FTIR), scanning electron microscopy (SEM), differential scanning calorimetry (DSC), and performance tests such as contact angle measurement, breakdown strength, arc resistance, dielectric constant tests, and a tower gap breakdown test. Results showed that APTES modification enhanced interface compatibility, leading to a uniform and dense microstructure. Compared with commercial polyurea (CPU) and commercial insulating sheath (CIS), HNTs/DPU exhibited superior performance: higher glass transition temperature (Tg) and thermal stability, excellent hydrophobicity, improved breakdown strength and dielectric constant, longer arc resistance time by blocking microcrack propagation, and optimal insulation effect at 4 mm coating thickness in the tower gap test with a significantly higher breakdown voltage. In conclusion, HNTs/DPU provides a new technical solution for transmission line insulation protection under extreme conditions, with comparative data demonstrating advancements over existing materials.

## 1. Introduction

Due to human activities such as the reduction of vegetation along transmission lines, transmission towers have gradually become habitats for birds, leading to a significant increase in bird-related hazards [1,2,3]. With the continuous advancement of the national ecological restoration strategy, the transmission corridors of State Grid are facing multiple environmental threats. According to statistics, among the 69 tripping accidents of 220–500 kV lines in Jiangsu Power Grid in 2024, 67% were caused by bird-related hazards, foreign objects, and wind deviation (galloping) faults [3]. Notably, many of these accidents occurred even after the deployment of various protective measures such as bird guards and insulating sleeves. In addition, extreme wind weather triggered by the global El Niño effect has been increasing year by year. Under extreme winds exceeding 30 m/s, the air gap of towers covered with traditional materials dielectric breakdown when shortened to less than 1 m—a wind speed that has gradually become normalized in Jiangsu Province, China. In 2024, foreign object entanglement faults caused by strong winds in the region increased significantly compared with previous years [4,5]. It is worth noting that Jiangsu’s unique agroforestry ecosystem and dense water network not only provide ideal habitats for birds but also increase the probability of foreign objects sources such as agricultural plastic films coming into contact with transmission lines. Such typical fault problems pose enormous challenges to the fault protection of transmission lines.

**Figure 1:**
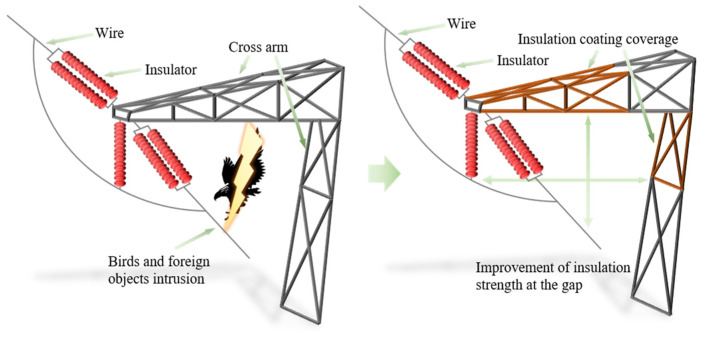
Schematic diagram of local insulation enhancement of transmission tower.

For 220 kV and above lines, some of the causes of the above faults include insufficient insulation margin of the tower air gap due to bird body short-circuiting, bird droppings intrusion, and conductor galloping, which is equivalent to a reduction in insulation distance, ultimately leading to air gap discharge breakdown of the tower [6,7]. Currently, an effective method is to coat insulating materials on the crossarm side of transmission towers to insulate the exposed metal parts near the grounding point, thereby reducing the occurrence of faults. As shown in Figure 1, solid insulation is applied to the surface of the transmission tower to form a combined insulation structure with air. This insulation enhancement method reduces the probability of accidents caused by bird droppings, bird bodies and other factors. However, the gap insulation enhancement technology for transmission lines faces multiple technical bottlenecks. Using traditional insulating sleeves to improve insulation strength has limited effect on enhancing insulation performance and is difficult to apply to high-voltage levels. Moreover, during long-term operation, there is a risk of moisture infiltration caused by seal failure, which can reduce dielectric properties by more than 30%, induce metal corrosion, further damage the dielectric system, and then lead to electrochemical corrosion of metal components and local overheating [8,9]. In addition, restricted by the structural characteristics of the tower, existing insulation measures can only cover the high-voltage area on the conductor side [6,7]. The ground potential side (such as crossarm connections and tower material intersections) has a high electric field distortion rate due to the presence of a large number of discharge tips, making it difficult for existing technologies to implement effective insulation protection, resulting in frequent gap discharge accidents.

Polyurea materials have garnered extensive attention in the field of insulation protection for the ground potential side of high-voltage transmission towers, attributed to their prominent characteristics including rapid curing, superior mechanical properties, and robust environmental aging resistance. Relevant research endeavors have yielded substantial technical accumulations, as elaborated below: In the realm of basic performance optimization, prior studies have investigated the effects of adjusting the molar ratio of polyurea resins. It was demonstrated that a rational increment in the content of amine compounds can mitigate defects within the curing system, reduce water absorption capacity, and concurrently enhance the hydrophobicity and electrical insulation performance of the coating. This finding provides a fundamental theoretical and experimental basis for the formulation refinement of polyurea materials. Regarding composite modification research, Jiaao Yan et al. incorporated Al_2_O_3_ powder as a reinforcing filler into polyurea coatings. Their results confirmed that an optimal dosage of Al_2_O_3_ can effectively improve the Shore hardness and corrosion resistance of the composite coating, thereby offering a viable technical pathway for the performance expansion of polyurea-based composites. In the exploration of application scenarios, Le Li et al. have systematically verified the insulation effectiveness of polyurea coatings when applied to the ground potential side of transmission towers. Specifically, they clarified that a polyurea layer with a thickness ranging from 3 to 5 mm can significantly enhance the breakdown voltage of the conductor-tower gap, furnishing critical technical parameters for practical engineering applications. Furthermore, existing research encompasses additional directions such as the optimization of polyurea coating application processes and the mechanistic analysis of insulation behavior under conventional electric field conditions. Collectively, these efforts have established a relatively comprehensive technical framework, laying a solid foundation for subsequent research in this field.

To address the above problems, the current research direction mainly focuses on developing high-performance composite insulating spray coatings with rapid curing (surface drying time < 15 min) to achieve adaptive coating of the complex structure of crossarms on the ground potential side, forming a continuous and dense insulating layer [10,11] and improving the dielectric properties of insulating materials. Among them, polyurea, as a new type of composite insulating spray material, has gradually demonstrated application potential in the field of power equipment protection in recent years. Coating polyurea on the low-voltage side can increase the characteristic breakdown voltage by 12.5% according to Luo et al. [12] and its mechanical properties are significantly superior to traditional epoxy resin coatings [13]. However, in the current research system of insulating spray polyurea, modification strategies are mostly limited to the simple filler doping of inorganic nanoparticles such as SiO_2_ and Al_2_O_3_, lacking targeted optimization design of the chemical structure of nanomaterials. This leads to insufficient interface compatibility between nanomaterials and the polyurea matrix, which seriously restricts the improvement of comprehensive material properties including dielectric properties. There is an urgent need to achieve this core breakthrough through the structural optimization of nanomaterials. In addition, for the complex electric field environment with insufficient equivalent gap distance of transmission lines, the adaptability research on the dielectric properties of polyurea materials has not yet formed a complete system, and relevant explorations still need to be further carried out around the correlation between nanomaterial modification and dielectric property improvement.

Despite the above progress, existing insulation enhancement strategies for transmission towers still face notable limitations. Traditional polymer-based coatings and insulating sleeves, including epoxy resins and silicone rubber materials, generally exhibit limited adaptability to complex tower geometries and insufficient long-term stability under distorted electric fields and moisture ingress. For polyurea-based insulating coatings, most reported modification approaches rely on the direct incorporation of inorganic fillers such as SiO_2_ or Al_2_O_3_, focusing primarily on mechanical reinforcement or corrosion resistance. However, insufficient interfacial compatibility between nanofillers and the polyurea matrix often leads to heterogeneous microstructures, restricted polarization efficiency, and limited improvement in dielectric performance. Moreover, systematic studies correlating nanofiller interfacial modification with dielectric behavior under reduced air-gap and high electric field distortion conditions remain scarce. Therefore, developing a polyurea-based insulating system with optimized interfacial interactions and enhanced dielectric response is still an urgent challenge for practical transmission line insulation protection.

This paper proposes a silane coupling agent-modified halloysite nanotube composite polyurea for preventing potential extreme wind deviation and bird-related hazards. Fourier transform infrared spectroscopy (FTIR), scanning electron microscopy (SEM), differential scanning calorimetry (DSC), contact angle measurement, and breakdown strength testing were used to characterize and test the prepared materials. A simulation platform was built to simulate the problem of equivalent air gap shortening in actual conditions. The test results show that the key dielectric performance indicators of the composite polyurea material, such as breakdown strength, dielectric constant, and arc resistance time, are significantly superior to existing commercial polyurea materials. It effectively breaks through the performance bottlenecks of traditional materials in complex electric fields and demonstrates promising application prospects. Compared with commercial polyurea (CPU) and insulating sheath (CIS), the HNTs/DPU composite developed in this study achieves a 59.2% increase in breakdown strength, a 35.5% increase in dielectric constant, and a 38.8% extension in arc resistance time, while maintaining excellent hydrophobicity (contact angle > 94°) and thermal stability (Tg = 62 °C). These improvements address the key performance limitations of existing materials in extreme environments.

## 2. Materials and Methods

### 2.1. Materials

Tolylene Diisocyanate (TDI), with a purity of 98%, was purchased from Aladdin (Shanghai, China); Methylenebis(p-phenyl isocyanate (MDI)), with a purity of 98%, was purchased from Aladdin; Polypropylene glycol bis(2-aminopropyl) ether (PPG-2000, PPG-400: number-average molecular weights Mn = 2000 and 400), respectively, with a purity of 99%, was purchased from Sigma–Aldrich (St. Louis, MO, USA); 2-Aminophenyl disulfide, with a purity of 98%, was purchased from Aladdin: *N*,*N*-Dimethylacetamide (DMAC), with a purity of 99%, was purchased from Aladdin. Commercial polyurea (CPU) and Commercial insulating sheath (CIS) were purchased from Shijiazhuang Weishiqi New Materials Co., Ltd. (Shijiazhuang, China) and Langfang Chang’ao Electric Power Technology Co., Ltd. (Langfang, China), respectively. Halloysite nanotubes (HNTs) were purchased from Xinlei Mineral Co., Ltd. (Shijiazhuang, China). APTES possesses bifunctional groups enabling dual reactions: one end, the triethoxysilyl group, can undergo hydrolysis and condensation with hydroxyl groups; the other end, the amino group, serves as a highly reactive functional site. It can react with various groups such as carboxyl and epoxy groups, facilitating subsequent functional modification, and the reaction conditions are mild and easy to control. Therefore, APTES is often used as a silanizing agent. Aminopropyltriethoxysilane (APTES), with a purity of 99%, was purchased from Aladdin. Anhydrous ethanol, with a purity of 99%, was purchased from Aladdin. Ammonia solution, analytical grade, was purchased from Aladdin.

### 2.2. Preparation of HNTs/DPU

**Figure 2:**
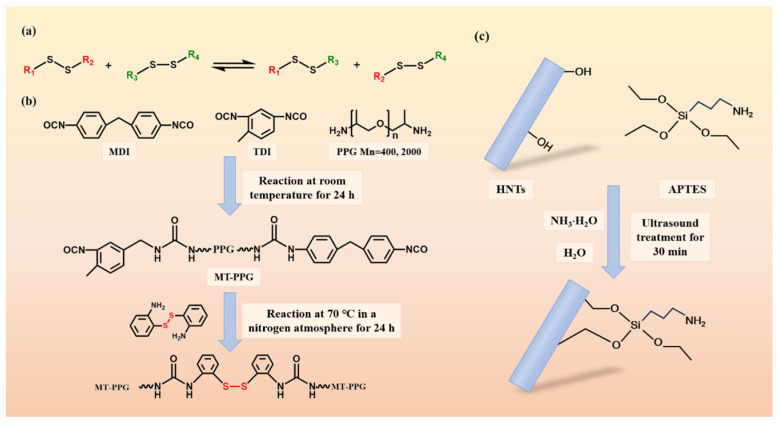
(**a**) Dynamic reaction mechanism of disulfide bonds: (**b**) Reaction process and structural schematic of disulfide-containing polyurea (DPU): (**c**) Schematic of APTES-modified HNTs reaction.

The disulfide exchange reaction of aliphatic disulfides requires triggering by ultraviolet light or temperature elevation to achieve dynamic exchange, while aromatic disulfides undergo dynamic exchange more easily. Therefore, based on the dynamic disulfide exchange reaction of disulfide bonds, this study prepared polyurea materials with dynamic covalent bonds to improve their comprehensive properties, and the preparation schematic is shown in Figure 2. Table 1 summarizes the experimental samples.

MDI and TDI were weighed at a molar ratio of 1.05:1.05 using a precision balance and added to 20 mL of DMAC solution. PPG (Mn = 2000) and PPG (Mn = 400) were weighed at a molar ratio of 1:1, and added dropwise to the mixed isocyanate solution of MDI and TDI. The mixture was continuously stirred under nitrogen atmosphere and reacted at room temperature for 24 h to obtain the isocyanate prepolymer solution MT-PPG1:1.

A total of 1.34 g of 2-aminophenyl disulfide was weighed using a precision balance and dissolved in DMAC solution. This solution was added dropwise to the isocyanate prepolymer solution over 30 min, followed by reaction at 70 °C for 24 h under nitrogen atmosphere to obtain the polyurea solution with dynamic disulfide bonds.

A total of 2 g of HNTs were added to 100 mL of anhydrous ethanol and stirred for 1 h. Then, 8 mL of APTES, 10 mL of distilled water, and 5 mL of ammonia solution were added, and the mixture was ultrasonicated for 30 min, followed by stirring at room temperature for 24 h. Unreacted APTES was removed by centrifugation at 6800 rpm for 10 min. The modified HNTs were obtained after drying in a vacuum oven.

A total of 0.5 g of modified HNTs was added to the polyurea solution, and the mixture was stirred for 2 h before being poured into a polytetrafluoroethylene (PTFE) mold. After drying in a vacuum oven for more than 2 h, the high-performance disulfide-containing polyurea composite HNTs/DPU was obtained. The overall yield (solid recovery) of the HNTs/DPU composite was 90.1%, calculated by a mass-balance approach based on the mass of the vacuum-dried composite (after solvent removal) divided by the theoretical total mass of solids added to the composite preparation step (DPU solids + HNTs). Because Step (b) is carried out in solution without isolating intermediate solids and Step (c) includes purification by centrifugation/washing to remove unreacted silane, isolated chemical yields for the individual sub-steps are not separately reported; therefore, only the overall solid recovery of the final composite is provided. In addition, DPU without HNTs and HNTs/DPU without APTES modification (UHNTs/DPU) were prepared for comparison.

### 2.3. Characterization and Test Methods

Fourier transform infrared spectroscopy (FT-IR, Nicolet Summit X, Thermo Fisher Scientific, Madison, WI, USA) was used to analyze the chemical structure of DPU. Before testing, the sample was ground, mixed with KBr, and pressed into pellets. The spectral scanning range was set to 400–4000 cm^−1^ with a resolution of 4 cm^−1^ and 32 cumulative scans.

Differential scanning calorimetry (DSC) was employed to characterize the glass transition behavior of polyurea samples. A NETZSCH DSC 3500 Sirius thermal analyzer was used. Approximately 8.0 ± 0.5 mg of the sample was accurately weighed and placed in a standard aluminum crucible, with the test conducted under the protection of high-purity nitrogen at a flow rate of 50 mL/min. The temperature program was set as follows: heating from 0 °C to 100 °C at a constant rate of 15 °C/min. The glass transition temperature (Tg) was determined as the midpoint of the transition curve for all samples to ensure consistency in comparison.

The surface wettability of DPU materials was evaluated by static contact angle testing to assess hydrophobicity. A standard contact angle measuring instrument (JC2000DM, Zhongchen, Shanghai) was used, with the test method described as follows: the sample to be tested was horizontally fixed on the test platform, and 4 μL of deionized water droplets (25 ± 1 °C) were deposited on the sample surface using a precision microsyringe. The droplet profile was captured in real-time by a high-speed camera and computer software, and the static contact angle θ was calculated by fitting with the Young–Laplace equation. To ensure data reliability, measurements were taken at 5 random positions on each sample surface, with 5 repetitions at each position. The arithmetic mean value was finally adopted as the static contact angle of the sample.

Field-emission scanning electron microscopy (SEM) at an acceleration voltage of 20 kV was used to investigate the microstructural characteristics of halloysite nanotubes and polyurea. After ion sputter coating with gold, the surface topology and phase distribution were observed in the secondary electron imaging mode.

In accordance with the industry standard HG/T3831-2006 [14], the prepared polyurea coatings were first fully dried in a blast drying oven. The test samples were cut into dimensions of 4 cm × 4 cm and then immersed in deionized water for 72 h. The weights of the coatings before and after immersion were measured separately. The water absorption rate (Wa) of the samples was calculated using Formula (1):

(1) $$W_{a}=\frac{m_{1}-m_{0}}{m_{0}}\times 100\%$$

In accordance with GB/T1408.1-2016 [15], the dielectric strength of the materials was measured in insulating oil using a sphere–sphere electrode system (diameter: 20 mm). The samples were prepared into discs with a diameter of 50 mm and a thickness of 2 mm, and an AC voltage was applied at a rate of 2 kV/s until breakdown. Each group of samples was tested 5 times repeatedly, and the breakdown field strength data were statistically analyzed using the two-parameter Weibull distribution.

In accordance with GB/T 1411-2002 [16], the arc resistance test of the samples was performed by applying an AC voltage to a tungsten rod electrode. After each test, the tungsten rod electrode was cleaned with anhydrous ethanol. The total time from the start of the test to the failure of the sample was recorded to characterize the arc resistance strength of the material.

The dielectric constant of the samples was tested using a WY2858-2 dielectric constant tester (Shanghai Precision Instruments Co., Ltd., Shanghai, China) at a test frequency of 50 Hz.

**Figure 3:**
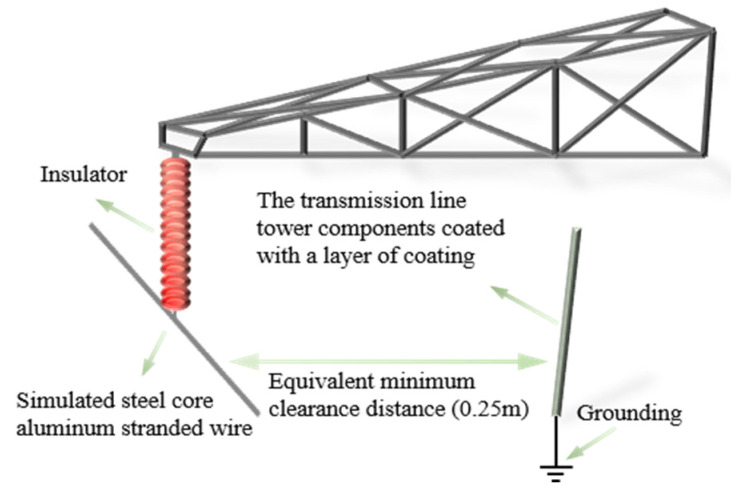
Schematic diagram of the tower gap breakdown test platform. The coating thickness δ was controlled by the number of spray passes and verified after drying using a digital thickness gauge and a vernier caliper at multiple positions. All measurements were repeated five times; results analyzed using one-way ANOVA with Tukey’s post-hoc test.

A tower gap breakdown test platform was built to evaluate the improvement effect of gap breakdown strength after local insulation, as shown in Figure 3. The test adopted a metal structure welded with Q235B steel to fully simulate the structural characteristics of the tower head-crossarm in actual transmission lines. An aluminum tube with a length of 2 m and a diameter of 20 mm was used to replace the conventional steel-cored aluminum strand, which was suspended under the crossarm through composite insulators. The ground electrode was a steel tube with a diameter of 20 mm, and its surface was sprayed with different thicknesses (δ) of CPU, CIS, and APTES-modified halloysite nanotube composite polyurea insulating coatings. The coating thickness δ was controlled by the number of spray passes and verified after surface drying using a digital thickness gauge (and a vernier caliper on test coupons) at multiple positions to ensure uniformity. The electrode spacing was set to the minimum air gap of 0.25 m for 110 kV in accordance with Clause 7.0.9 of GB 50545-2010 “Code for Design of 110~750 kV Overhead Transmission Lines”. The power frequency voltage was controlled by a voltage generator at a boosting rate of 2 kV/s. The test environment conditions were (25 ± 2 °C) and relative humidity (50 ± 5%). The breakdown voltage was measured by the step-up method, with 10 valid tests repeated for each group of samples. The voltage value at the moment of breakdown was recorded, and a Weibull distribution diagram was drawn for statistical analysis [17].

## 3. Results and Discussion

### 3.1. The Microstructure of HNTs/DPU

#### 3.1.1. FTIR Analysis (Chemical Structure)

Fourier transform infrared spectroscopy (FT-IR) is an important molecular structure characterization technique that can analyze the type of chemical bonds and molecular structural characteristics by detecting the transition of molecular vibrational energy levels. Through the characteristic absorption peaks generated by different chemical bonds at specific wavenumbers, it can not only identify the category of compounds but also be used for quantitative analysis.

**Figure 4:**
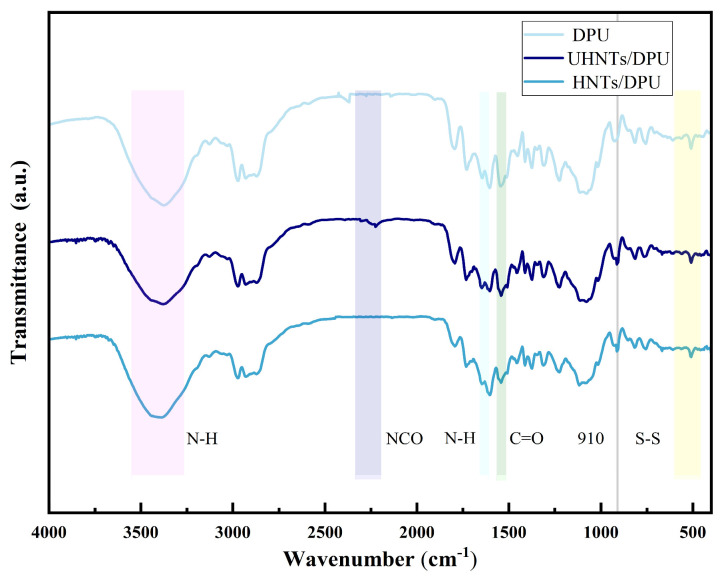
FTIR spectra of the polymer matrix (DPU) and DPU-based composites containing unmodified and APTES-modified HNTs (4000–500 cm^−1^). The shaded regions highlight: (i) the N-H stretching region (3000–3500 cm^−1^, pink area), (ii) the -NCO band region (2240–2280 cm^−1^, purple area), (iii) the C-O vibration region (1500–2000 cm^−1^, green area), and (iv) the S-S vibration region (around 500 cm^−1^, yellow area). The Si-O/Al-O vibration region (linked to the 910 cm^−1^ band) is used to compare UHNTs and modified HNTs; the disappearance of the -NCO band in cured samples indicates complete consumption of isocyanate groups during curing.

Figure 4 shows the FT-IR spectra of different HNTs/DPU composites [8]. It can be seen from the figure that in the range of 2240~2280 cm^−1^, the characteristic peak generated by the stretching vibration of the NCO group is almost not observed. This indicates that the -NCO group gradually reacts with the -NH group and forms urea linkages, indicating the successful curing of the DPU network. The absorption peak near 3413 cm^−1^ corresponds to the N-H stretching vibration, while the peaks near 2854 cm^−1^ and 2932 cm^−1^ are attributed to the symmetric and asymmetric C-H stretching vibrations, respectively. The absorption peak around 1104 cm^−1^ is the C-O-C stretching vibration, and the peak near 520 cm^−1^ corresponds to the stretching vibration of the disulfide bond (-S-S-) [18]. In addition, after the introduction of unmodified HNTs, some residual NCO groups seem to exist, which may indicate that the direct addition of HNTs may have a certain inhibitory effect on the synthesis of DPU. However, the introduction of APTES-modified HNTs into DPU did not significantly affect the participation of NCO in DPU synthesis, as evidenced by the disappearance of the -NCO group in HNTs/DPU. This suggests that the HNTs/DPU nano-insulating material may have higher chemical structure stability. FT-IR observations also show that after the introduction of HNTs, absorption peaks of varying intensities appear in the range of 3750~4000 cm^−1^ and at 910 cm^−1^, which may be caused by the characteristic peaks of HNTs [9].

#### 3.1.2. SEM Analysis (Morphology and Dispersion)

**Figure 5:**
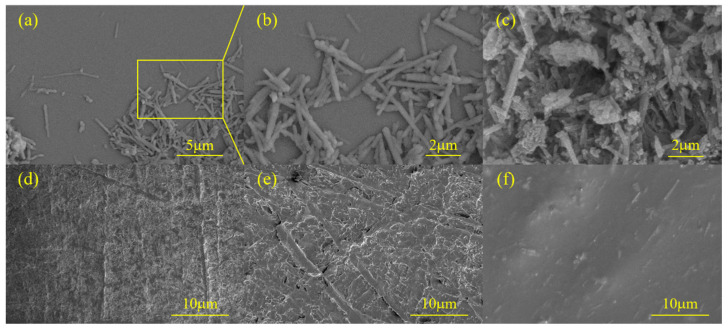
SEM images of (**a**,**b**) unmodified HNTs, (**c**) APTES-modified HNTs, (**d**) DPU matrix, (**e**) UHNTs/DPU composite, and (**f**) APTES-modified HNTs/DPU composite. All SEM images were taken under the same accelerating voltage and magnification for comparison. Surface morphology observed by SEM (qualitative only).

Scanning electron microscopy (SEM) images (Figure 5a–f) were used to observe the morphological changes of HNTs before and after modification, as well as the surface morphology evolution of DPU composites. As shown in Figure 5a,b, the surface of unmodified HNTs exhibits a rod-like tubular structure. After APTES modification (Figure 5c,d), blocky or flaky deposits can be observed on the HNTs surface, which may be due to the agglomeration of part of the APTES after modification [19]. It can be observed that the nanotubular structure of HNTs is not damaged by APTES modification and remains overall stable.

The DPU matrix exhibits a relatively homogeneous surface morphology (Figure 5d). This observation is consistent with the microphase-separated nature of polyurea systems, where the phase morphology can be influenced by the soft/hard segment ratio and the mobility of the soft segment (e.g., PPG-2000 vs. PPG-400) [20,21]. However, SEM primarily provides qualitative morphological information; therefore, mechanistic explanations regarding segment enrichment or phase continuity are discussed here only as possible interpretations. After incorporating UHNTs (Figure 5e), small aggregates can be observed, which may be related to insufficient interfacial compatibility. In contrast, the APTES-modified HNTs/DPU sample (Figure 5f) shows a more uniform filler dispersion with fewer visible agglomerates, suggesting improved interfacial interaction after silane treatment. SEM observations provide only qualitative morphology; quantitative surface roughness would require profilometry or AFM [22].

#### 3.1.3. Thermal Analysis (DSC/Tg, Thermal Stability)

**Figure 6:**
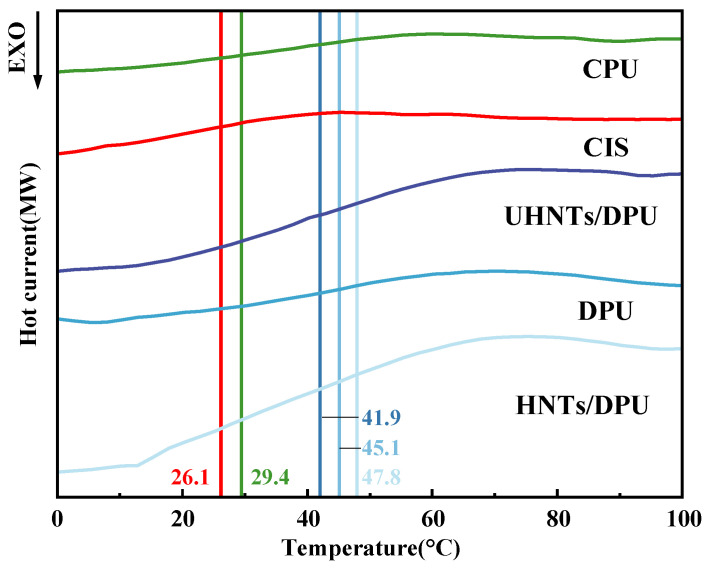
DSC curves of nanocomposites and common commercial materials.

The glass transition temperature (Tg) obtained by DSC reflects the mobility of polyurea molecular chains and the degree of microscopic phase separation [23]. As shown in Figure 6, in DPU without HNTs, PPG-2000 and PPG-400 are mixed in equal proportions; theoretically, the average molecular weight of the soft segment composed of these two PPGs would be between the two individual molecular weights [24]. At this time, the degree of microphase separation between the soft segment and the hard segment is reduced, which may restrict the movement of some soft segments. After adding unmodified HNTs (UHNTs/DPU), the Tg of DPU decreases, which may be attributed to the destruction of hydrogen bonds in some DPU long chains caused by the introduction of nanofillers. Although the disulfide bonds in the chain extender can enhance intermolecular forces, their direct impact on Tg in the hard segment is limited, so the introduction of HNTs becomes the main factor directly affecting the Tg of the material [25]. Compared with APTES-modified HNTs, the closer combination with DPU leads to an increase in Tg. In addition, we also compared the Tg of commonly used commercial insulating materials CIS and CPU. The low glass transition temperature of CIS indicates its high flexibility, which helps the insulating sleeve adapt to electrical equipment of various shapes [26]. However, in complex transmission line scenarios, heat generation caused by distorted electric fields and heat accumulation due to high temperatures in summer may accelerate the degradation of the insulating sleeve. It is worth noting that HNTs/DPU no longer only relies on the hydrogen bonds of polyurea itself to maintain material stability. The presence of modified HNTs significantly increases the glass transition temperature of polyurea, showing much higher stability than CPU [27].

### 3.2. The Hydrophobic Properties of HNTs/DPU

**Figure 7:**
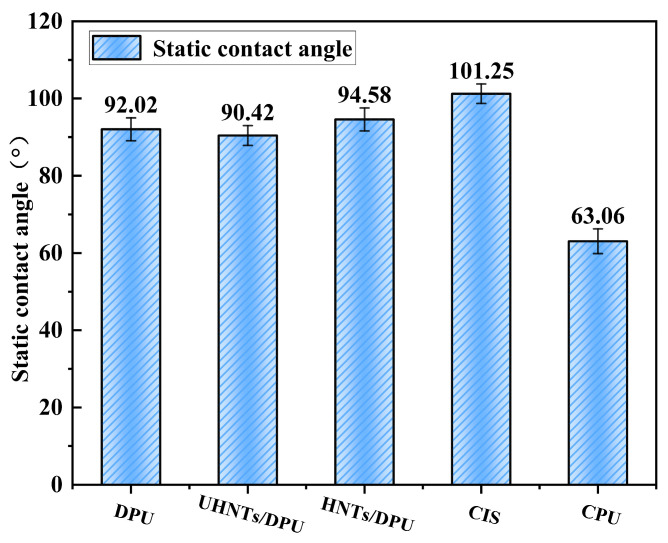
Static water contact angles of DPU-based nanocomposites and commercial reference materials. Each value represents the mean of five measurements at different positions on each specimen; error bars denote the standard deviation (SD). Statistical analysis (pairwise *t*-test) showed no significant difference between HNTs/DPU and CIS (*p* > 0.05), while HNTs/DPU was significantly higher than CPU (*p* < 0.01).

The contact angle directly reflects the surface energy of the material and is also an important indicator for the application of nano-insulating materials in the extreme environment of transmission lines. Surface energy is affected by the hydrophobicity of soft segments (PPG) and nanofillers [28]. As shown in Figure 7, due to the presence of more hydrophobic repeating units (-CH_2_-CH(CH_3_)-O-) in long-chain PPG-2000, the enrichment of methyl groups (-CH_3_) reduces the surface energy of the material. Meanwhile, PPG-2000 is more likely to migrate to the surface and stably exist there, masking the internal polar urea bonds, thus making the contact angle exhibit hydrophobicity (>90°) [29]. After adding HNTs, the surface state of DPU undergoes significant changes. The increase in roughness exposes more polar groups, which may lead to an increase in the surface energy of the material and thus a decrease in the contact angle.

It should be noted that SEM provides qualitative surface morphology only and does not yield quantitative roughness parameters (e.g., Ra/Rq). Quantitative roughness evaluation would require profilometry or AFM. Therefore, roughness-related interpretations in this work are discussed cautiously and are limited to what can be supported by the SEM features together with the measured contact-angle results (Figure 7).

However, the modified HNTs show a good surface morphology, enabling the surface to maintain the CASSIE state and thus a high apparent contact angle—this may be one of the reasons for the improved hydrophobic angle [30]. Compared with CIS, HNTs/DPU still has a certain gap in the hydrophobic angle, but CIS might also be in the same statistical bracket as the DPU-based samples. Since the detailed formulation and possible surface treatments of the commercial insulating sheath (CIS) are proprietary and were not characterized in this work, we avoid attributing its contact-angle behavior to a specific chemical treatment. Instead, we discuss CIS wettability based on the measured values in Figure 7 (mean ± SD) and present the comparison in a data-driven manner. In addition, the lack of stable enrichment of long-chain segments on the surface of CPU molecules indirectly reduces the contact angle—this is the only measurement that visibly differs, so this explanation is more reliable. It is worth noting that the contact angle of HNTs/DPU is much higher than that of currently commercial polyurea insulating materials, showing strong application prospects.

**Figure 8:**
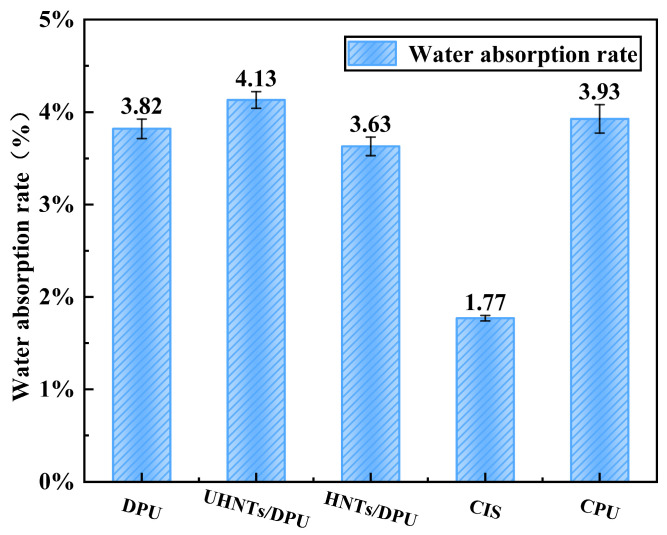
Water absorption rates of DPU-based nanocomposites and commercial reference materials after 24 h immersion. Each value represents the mean of three independent specimens; error bars denote SD. SD denotes standard deviation; no significant difference was observed between HNTs/DPU and DPU (*p* > 0.05), whereas UHNTs/DPU showed significantly higher water absorption (*p* < 0.05). Each value represents the mean ± SD of three specimens; no significant difference (*p* > 0.05) between HNTs/DPU and DPU.

Figure 8 shows the water absorption rate of different nanocomposites and common commercial materials. Statistical analysis of contact angle and water absorption data confirmed that the hydrophobicity and water resistance of HNTs/DPU were significantly superior to commercial polyurea (CPU) (*p* < 0.05). Since the molecular weight of PPG-2000 is significantly higher than that of PPG-400, its long-chain structure will enhance the hydrophobicity of the soft segment. The increased proportion of long-chain hydrophobic components in the soft segment reduces the overall water affinity of the material. In addition, the microphase separation behavior of polyurea materials is significantly affected by the length of the soft segment. The long-chain structure of PPG-2000 is more likely to form a continuous soft segment phase region, while the hard segments aggregate into a dispersed phase. This more complete microphase separation structure limits the diffusion path of water molecules in the polar hard segments, resulting in a lower water absorption rate compared with CPU [20]. After adding unmodified HNTs, the water absorption rate increases, which may be due to the poor compatibility between HNTs and DPU, leading to potential cracks or pores that make the material more prone to water absorption—this is also confirmed by the SEM images (Figure 5e). In contrast, HNTs modified with APTES can significantly enhance the interfacial bonding force between nanofillers and the polymer, making it difficult for moisture to penetrate and thus reducing the water absorption rate [28].

### 3.3. The Dielectric Properties of HNTs/DPU

**Figure 9:**
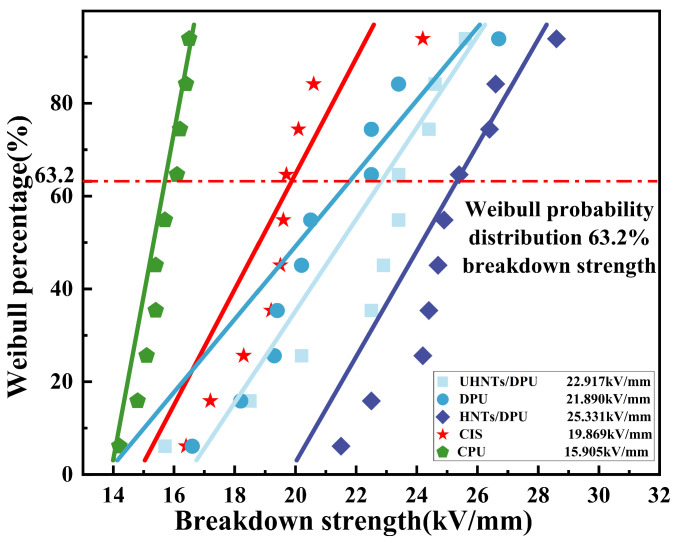
Breakdown strength of nanocomposites and common commercial materials.

Breakdown strength is a crucial indicator for evaluating nano-insulating materials. The breakdown strength of DPU is also closely related to the degree of microphase separation [31]. In the experiment, test samples are prepared by spraying insulating materials. After the materials are surface-dried, a vernier caliper is used to ensure the materials have sufficient and uniform thickness. As shown in Figure 9, the breakdown strength of CPU is 15.91 kV/mm. Its low glass transition temperature may cause a sudden change in the dielectric constant of the internal cross-linked network in an AC electric field, thereby inducing electric field distortion. In contrast, the cross-linked network of DPU regulated by dynamic disulfide bonds has a more uniform free volume distribution, which reduces the space charge density and results in higher breakdown strength. With the introduction of unmodified HNTs into DPU, the breakdown voltage increases to a certain extent. This phenomenon may be attributed to the large dielectric constant of HNTs themselves [32]. In the internal network of DPU, such nanofillers with a large aspect ratio can effectively block the further development of electrical trees. This effect compensates for the defects caused by the introduction of unmodified HNTs, so the difference in breakdown strength compared with DPU without HNTs is not significant. After the introduction of modified HNTs, the breakdown strength of DPU shows a substantial improvement. This may be because the modified HNTs and DPU tend to form a more uniform mixed phase of soft and hard segments, leading to a more uniform distribution of polar urea bonds (-NH-CO-NH-). The dielectric properties of DPU are largely affected by urea bonds: their uniform distribution can avoid excessive phase mixing and prevent uneven dispersion of hard segments caused by overly long soft segments, thereby forming a moderate microphase separation structure and reducing electric field distortion. At this point, the cross-linking density reaches an optimal value, which not only ensures the integrity of the polyurea cross-linked network but also maintains the mobility of the chain segments. During the breakdown process, it is plausible that the inherent charge traps of the material and those introduced by HNTs may capture free electrons more effectively, potentially forming a charge trap network. This interaction could delay the breakdown process and enhance the characteristic breakdown voltage, a trend that aligns with observations in polyurethane/organoclay systems reported in the literature [33]. Consequently, the dielectric properties are significantly improved compared to CIS.

**Figure 10:**
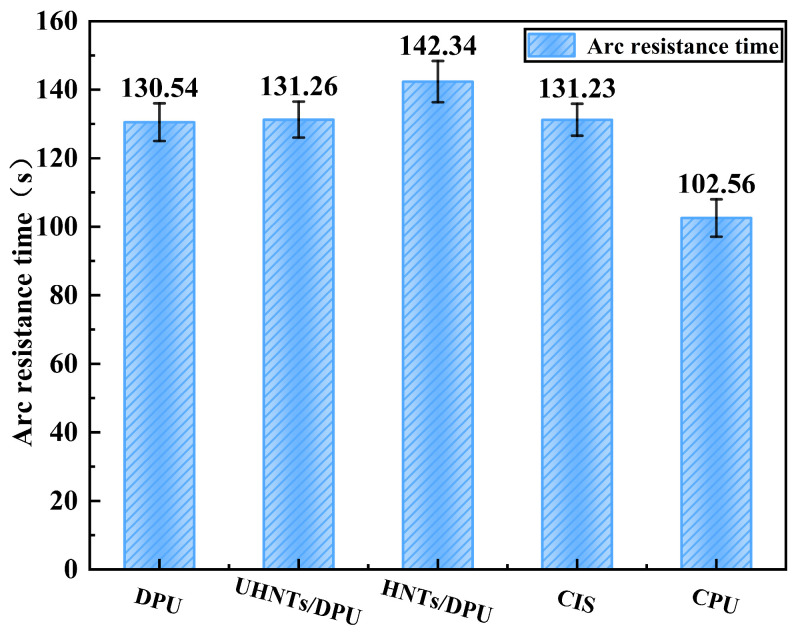
Arc resistance time of DPU-based nanocomposites and commercial reference materials. Each value represents the mean of five measurements; error bars denote SD. Statistical significance was evaluated using one-way ANOVA with Tukey’s multiple-comparison test.

Figure 10 shows the arc resistance duration of nano-insulating materials. The test results indicate that modified HNTs significantly enhance the arc resistance of DPU, which may be related to HNTs blocking the propagation of arc-induced microcracks. Its high cross-linking density inhibits the arc development process, and the arc resistance time even exceeds that of CIS. In contrast, CIS relies on inorganic fillers and surface modification to disperse arc energy but lacks a dynamically cross-linked internal structure to maintain material stability, making it prone to performance degradation due to high temperatures under long-term arc action [9]: CPU is limited by its low glass transition temperature and low AC breakdown strength, resulting in the lowest arc resistance.

**Figure 11:**
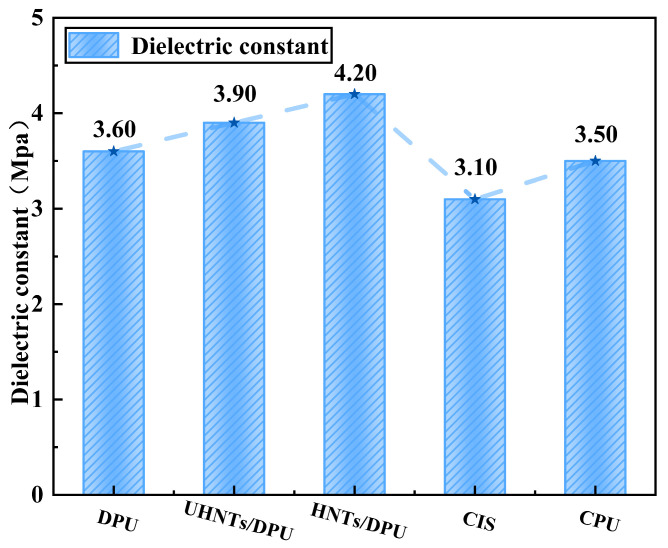
Dielectric constants of nanocomposites and common commercial materials.

Dielectric constant is an important reference for the dielectric performance design of nano-insulating materials. Figure 11 shows the dielectric constants of the materials under a 50 Hz alternating electric field. It can be seen from the figure that the dielectric constants of all tested polyureas are higher than that of CIS, which may be due to the presence of urea bonds with high dipole moments in polyurea [21]. After the introduction of HNTs, the dielectric constants of DPU all increase. As a type of nanoclay material, HNTs contain polar bonds such as siloxane bonds and aluminoxane bonds in their crystal structure. These bonds are prone to dipole polarization under alternating electric fields, leading to a high dielectric constant. When HNTs are introduced into the DPU matrix, their high dielectric properties increase the overall dielectric constant of the composite, regardless of modification. In addition, the DPU with APTES-modified HNTs has the highest dielectric constant. This may be because APTES modification greatly improves the dispersibility and interfacial bonding force of HNTs in the DPU matrix, thereby more effectively exerting the dielectric properties of HNTs and changing the microscopic polarization mechanism of the composite. A tightly bonded interface layer with different dielectric properties is formed between the modified HNTs and the DPU matrix. Due to the differences in dielectric constant and conductivity between the two phases, free charges accumulate at this uniform interface under alternating electric fields, resulting in strong interfacial polarization. This polarization makes a significant contribution to the dielectric constant.

### 3.4. The Performance of Gap Withstand Voltage

Based on the above experimental results, it can be found that the comprehensive performance of DPU with APTES-modified HNTs is the most excellent. Therefore, HNTs/DPU was selected as the optimal nano-insulating material for the gap breakdown voltage experiment. It was uniformly coated on the tower material by spraying using a Graco GHX-2 polyurea sprayer for testing.

**Figure 12:**
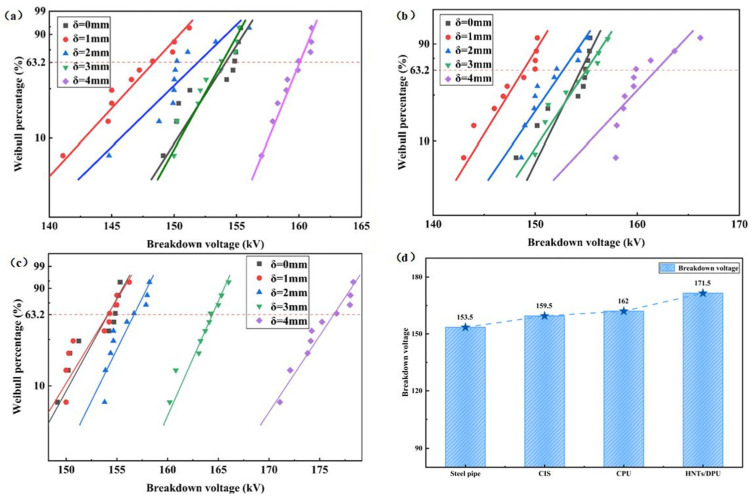
Weibull distribution of gap breakdown voltage for tower materials coated with (**a**) CIS, (**b**) CPU, (**c**) HNTs/DPU at different coating thicknesses, (**d**) gap breakdown voltage values of different insulating materials at 4mm coating thickness.

Figure 12 shows the Weibull distribution of the minimum gap breakdown voltage with the variation of three different insulating coating thicknesses at a minimum gap distance of 0.25 m. The experimental results indicate that for the bare tower material alone, the characteristic breakdown voltage is 147.5 kV. When the thicknesses of CPU and CIS coatings are small, the characteristic breakdown voltage of the material even drops below that of the uncoated tower material. This phenomenon may occur because the thin coating cannot fully cover the micro-rough field of the tower material surface, leading to the formation of air gaps or local electric field distortion. In contrast, the DPU with APTES-modified HNTs has a higher degree of cross-linking and a denser internal structure, resulting in better coating performance. Even at a small spray thickness, it can still achieve a certain improvement in insulation performance. As the coating thickness gradually increases, the gap breakdown voltage of CPU and CIS shows a rebound increase. This may be because the increased thickness extends the discharge path. Eventually, all three materials achieve the best insulation effect at a thickness of 4 mm. Figure 12d shows the change in breakdown voltage of the three different insulating materials compared with the uncoated one at a thickness of 4 mm. It can be seen from the figure that the improvement effect of HNTs/DPU is significantly superior to the other two insulating coatings, exhibiting excellent insulation protection performance. Furthermore, it represents a 12% increase compared with the breakdown voltage measured without insulation coating.

## 4. Conclusions

This study focuses on the insulation protection of transmission lines under extreme environmental conditions and complex fault scenarios. By designing a dynamic polyurea matrix and modifying nanoclay, the HNTs/DPU composite insulating material was prepared, and a series of characterizations and performance tests were conducted. The main conclusions are as follows:

With disulfide-containing dynamic polyurea (DPU) as the matrix and APTES-modified halloysite nanotubes (HNTs) as the reinforcing phase, the prepared HNTs/DPU composite exhibits excellent interfacial compatibility in microstructure. APTES modification enables more uniform dispersion of HNTs in DPU, and the composite surface is dense. Moreover, its glass transition temperature (Tg) is higher than that of commercial polyurea (CPU), indicating significant enhancement in molecular chain rigidity and thermal stability. Due to the long-chain structure and compact microstructure of PPG-2000, HNTs/DPU exhibits extremely high hydrophobicity and water resistance; its breakdown strength and dielectric constant are superior to those of traditional materials, attributed to the uniform distribution of polar urea bonds, the charge trap effect of HNTs, and strong interfacial polarization; its arc resistance time is longer than that of commercial insulating sheath (CIS) and CPU, benefiting from high cross-linking density and the barrier effect of HNTs on microcracks; in the tower gap breakdown test, the optimal insulation enhancement effect is achieved at a coating thickness of 4 mm, effectively solving the problem of insufficient insulation margin caused by air gap shortening under extreme winds.

Table 2 summarizes a comparative evaluation of key properties between the HNTs/DPU composite developed in this study and representative commercial and literature-reported polyurea-based materials. Compared with commercial polyurea (CPU) and commercial insulating sheath (CIS), HNTs/DPU exhibits a more balanced and comprehensive performance profile, including higher breakdown strength, larger dielectric constant, longer arc resistance time, and increased glass transition temperature. In addition, surface-related properties such as static contact angle and water absorption, which are critical for long-term operation under humid and contaminated transmission-line environments, are significantly improved for HNTs/DPU. It should be noted that for many previously reported polyurea-based composites, including Al_2_O_3_ and SiO_2_ filled systems, tower-gap breakdown voltage, surface wettability, and moisture resistance are rarely reported. As a result, these data are not available for direct comparison. Nevertheless, the expanded comparison in Table 2 highlights that the present HNTs/DPU system provides a more comprehensive set of experimentally validated dielectric, interfacial, and engineering-relevant properties, which are essential for insulation enhancement of transmission towers under complex electric field conditions.

In summary. This study innovatively combines dynamic disulfide bond design with nanoclay modification. The prepared HNTs/DPU composite overcomes the bottlenecks of traditional materials such as poor interfacial compatibility and limited dielectric properties. Additionally, it has good sprayability and adaptability to complex tower structures, providing a high-performance and reliable insulating material option for the safe operation of power grids under extreme environmental conditions and exhibiting broad application prospects in the field of power equipment insulation protection.

## Figures and Tables

**Table 1:** Summary Table of Experimental Samples.

Material Type	Polymer Matrix	HNTs Loading (g)	APTES Modification	Remarks
DPU	DPU	0	-	Pure DPU without HNTs
UHNTs/DPU	DPU	0.5	NO	Unmodified HNTs/DPU composite
HNTs/DPU	DPU	0.5	YES	APTES-modified HNTs/DPU composite
CPU	Commercial Polyurea	0	-	Commercial reference sample
CIS	Commercial Insulating Sheath	-	-	Commercial reference sample

**Table 2:** Comparative table of properties between HNTs/DPU and other published polyurea-based materials.

Material Type	Arc Resistance Time (s)	Dielectric Constant	Breakdown Strength (kV/mm)	Tg (℃)	Tower-Gap Breakdown Voltage (kV)	Static Contact Angle (°)	Water Absorption (%)
HNTs/DPU (this study)	142.34	4.20	25.33	62	171.5	94.58	3.63
Commercial Polyurea (CPU)	102.56	3.50	15.91	45	162.0	63.06	3.93
Commercial Insulating Sheath (CIS)	131.23	3.10	19.87	38	159.5	101.25	1.77
Al_2_O_3_/Polyurea Composite	118.7	3.80	20.5	51	N/A	N/A	N/A
SiO_2_/Polyurea Composite	125.3	3.90	21.2	48	N/A	N/A	N/A

Note: N/A indicates that the corresponding data were not reported in the present study or in the referenced literature under comparable test conditions. Tower-gap breakdown voltages were extracted from Figure 12d at a coating thickness of 4 mm.

## Data Availability

The original contributions presented in this study are included in the article. Further inquiries can be directed to the corresponding author.
